# Targeting Tissue Lipids in Age-related Macular Degeneration

**DOI:** 10.1016/j.ebiom.2016.02.003

**Published:** 2016-02-02

**Authors:** Rajendra S. Apte

**Affiliations:** Washington University School of Medicine, 660 South Euclid Avenue, Box 8096, St. Louis, MO 63110, United States

**Keywords:** Lipids, AMD, Retina, Eye, Macular degeneration, Cholesterol, Inflammation, macrophage

Age-related macular degeneration (AMD) is the leading cause of blindness among people over 50 years of age in the industrialized world (van Leeuwen et al., 2003). A cardinal clinical feature of the disease is extracellular deposition of lipid-rich deposits underneath the retinal pigmented epithelium (RPE) called drusen, which contain high amounts of esterified and unesterified cholesterol (Curcio et al., 2005). In AMD, lipid accumulation can also be observed as sub-retinal drusenoid deposits or as thickening of the Bruch's membrane, an elastin-rich layer that separates the RPE from the choriocapillaris. Although drusen themselves do not always affect vision, they typically precede advanced AMD, which is associated with vision loss. Advanced AMD occurs in two forms that can co-exist: a) geographic atrophy (GA) associated with loss of RPE cells and b) choroidal neovascularization (CNV) characterized by proliferative, anomalous growth of blood vessels underneath the retina that leads to hemorrhage and fibrosis (Fig. 1). Both GA and CNV ultimately cause vision loss due to photoreceptor cell death.

Recent studies demonstrate that lipid deposits in AMD serve as a nidus for disease progression (Sene and Apte, 2014, Sene et al., 2013, Sene et al., 2015). There are multiple mechanisms by which drusen and other extracellular lipids get deposited underneath the retina and accelerate disease pathogenesis: i) age-associated impairment of cholesterol homeostasis and reverse cholesterol transport (RCT) by macrophages and RPE, ii) lipid mediated RPE dysfunction and iii) activation of the innate immune response including aberrant macrophage polarization, activation of the complement cascade and accumulation of membrane attack complexes (MAC) around drusen and in the choroid (Mullins et al., 2014).

Therefore, reducing the lipid burden in AMD without causing RPE atrophy or stimulating CNV is a desirable outcome and merits investigation. Previous interventions such as laser to reduce drusen have not been successful (Virgili et al., 2015). In this issue of *EBioMedicine*, Vavvas et al. report anatomic and visual outcomes of a prospectively designed, non-randomized lipid lowering strategy in patients with AMD (Vavvas et al., 2016). They demonstrate a quantitative reduction in drusen volume in 10 of 23 patients placed on high dose, oral atorvastatin without significant change in visual acuity. Although baseline cholesterol levels of participants were reported, lipid profiling including lipid class and lipoprotein particle size information was not reported. These measurements may have provided key insights into why there was no correlation between drusen reduction and serum lipid response to atorvastatin in this study. Carefully collected baseline lipid metrics will be essential in future studies.

This study is very relevant since prior epidemiological studies have reported conflicting results regarding the efficacy of statins in AMD progression (Maguire et al., 2009). The report by Vavvas et al. illustrates the complexity of lipid modulation as a therapeutic strategy (Vavvas et al., 2016). There are several unanswered questions: a) Is the natural history of disease always associated with an increase in drusen volume? A matched control group would be necessary to make definitive conclusions; b) Does an increase in drusen volume correlate with increased risk for developing GA or CNV?; c) Is there an association between a reduction in macular drusen and RPE atrophy as has been previously reported?; d) What is the optimal time and dose of treatment to assess therapeutic response and side effects so that the risk to benefit ratio can be reduced? These questions mandate large, randomized prospective studies. As such, we should exercise caution in interpreting the results of this small, prospective cohort.

We may be on the cusp of potential breakthroughs in lipid-targeted treatment of AMD. Genome wide association studies (GWAS) of AMD patients have demonstrated polymorphisms in multiple genes involved in cholesterol homeostasis (Neale et al., 2010). Some examples are ATP-binding cassette transporter member 1 (ABCA1), which plays a key role in RCT, and genes such as hepatic lipase (LIPC) and cholesteryl ester transfer protein (CETP), which are involved in regulating HDL. Of interest, polymorphisms identified through GWAS suggest that lipid regulation and deposition in the eye is complex and may require targeted strategies beyond modulation of a single lipid class. Such strategies may include microRNA (miR) silencing approaches that target specific miRs, such as miR33, to regulate cholesterol synthesis and RCT; Liver X receptor (LXR) agonists to enhance RCT; and CETP modulators to alter serum HDL and particle size (Sene and Apte, 2014). These novel treatment approaches may synergize with other existing strategies such as statins to reduce cholesterol synthesis. Given the complexity of cholesterol homeostasis, each of these pathways will need careful investigation for efficacy and safety in animal models and randomized clinical trials that are adequately powered to probe these associations. Individualized therapy may be required depending on the pathways affected in each individual. It is biologically plausible that different pathways lead to drusen formation even though the ultimate phenotype is similar. If successful, this approach will usher in a new era in AMD therapy where disease can be targeted at an earlier stage.

## Figures and Tables

**Fig. 1:**
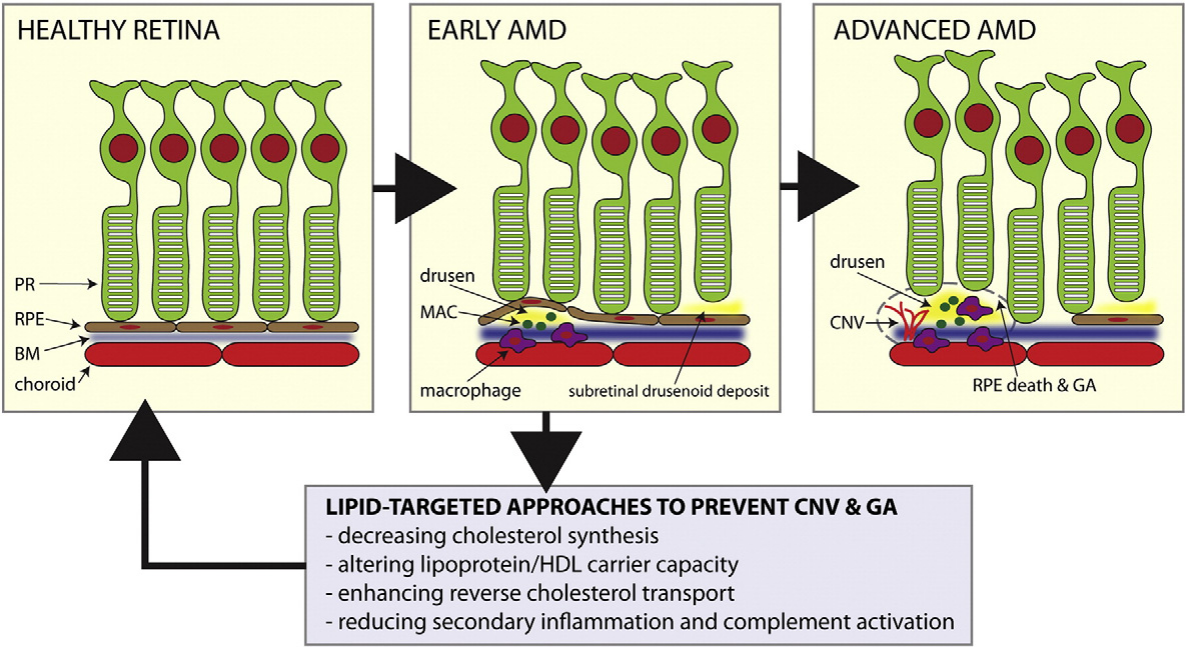
**Natural history of age**-**related macular degeneration** (**AMD**) **and potential role of lipid targeted therapies**. Early AMD is characterized by lipid rich drusen, sub-retinal drusenoid deposits, and thickening of Bruch's membrane (BM) along with abnormalities in the retinal pigmented epithelium (RPE). Macrophage-mediated inflammation, complement activation as manifested by the deposition of membrane attack complexes (MAC), and RPE dysfunction lead to advanced disease characterized by choroidal neovascularization (CNV) and geographic atrophy (GA). Potential therapeutic approaches to prevent complications of AMD are highlighted. PR = photoreceptor neurons.
